# Comparative two time-point proteome analysis of the plasma from preterm infants with and without bronchopulmonary dysplasia

**DOI:** 10.1186/s13052-019-0676-0

**Published:** 2019-08-24

**Authors:** Magdalena Zasada, Maciej Suski, Renata Bokiniec, Monika Szwarc-Duma, Maria Katarzyna Borszewska-Kornacka, Józef Madej, Beata Bujak-Giżycka, Anna Madetko-Talowska, Cecilie Revhaug, Lars O. Baumbusch, Ola D. Saugstad, Jacek Józef Pietrzyk, Przemko Kwinta

**Affiliations:** 1 Department of Pediatrics, Jagiellonian University Medical College, Cracow, Poland; 2 Chair of Pharmacology, Jagiellonian University Medical College, Cracow, Poland; 3 Neonatal and Intensive Care Department, Medical University of Warsaw, Warsaw, Poland; 4 Department of Medical Genetics, Jagiellonian University Medical College, Cracow, Poland; 5 Department of Pediatric Research, Oslo University Hospital, Oslo, Norway; 6 University of Oslo, Oslo, Norway

**Keywords:** Prematurity, Bronchopulmonary dysplasia, Proteome, Plasma

## Abstract

**Background:**

In this study, we aimed to analyze differences in plasma protein abundances between infants with and without bronchopulmonary dysplasia (BPD), to add new insights into a better understanding of the pathogenesis of this disease.

**Methods:**

Cord and peripheral blood of neonates (≤ 30 weeks gestational age) was drawn at birth and at the 36th postmenstrual week (36 PMA), respectively. Blood samples were retrospectively subdivided into BPD(+) and BPD(−) groups, according to the development of BPD.

**Results:**

Children with BPD were characterized by decreased afamin, gelsolin and carboxypeptidase N subunit 2 levels in cord blood, and decreased galectin-3 binding protein and hemoglobin subunit gamma-1 levels, as well as an increased serotransferrin abundance in plasma at the 36 PMA.

**Conclusions:**

BPD development is associated with the plasma proteome changes in preterm infants, adding further evidence for the possible involvement of disturbances in vitamin E availability and impaired immunological processes in the progression of prematurity pulmonary complications. Moreover, it also points to the differences in proteins related to infection resistance and maintaining an adequate level of hematocrit in infants diagnosed with BPD.

## Background

Despite indubitable improvements in neonatal care, bronchopulmonary dysplasia (BPD) remains a most frequent, adverse outcome of prematurity [1]. Until now, the pathophysiology of BPD has not been completely understood, and there are only a few effective, preventive and targeted treatment strategies for this disease [2]. The known BPD risk factors include: altered lung development in utero, arrest of normal alveolarization and lung vascular formation due to preterm birth, ventilator- and oxygen-induced injury to the immature lung, nutritional deficits impairing lung maturation, inflammatory response and genetic susceptibility [2, 3]. However, despite being in a group of high-risk BPD development, not all premature infants suffer from this lung complication [4]. Proteomics may be useful in developing much needed early biomarkers of lung injury, elucidating pathological pathways and determining protein abundance changes associated with disease progression, which may contribute to the development of new treatment strategies. It may also be helpful in explaining the susceptibility of some preterm newborns to BPD. Up to now, limited research has identified several proteins, like sialic acid-binding Ig-like lectin 14, Basal Cell Adhesion Molecule and Angiopoietin-like Protein 3, in which altered levels in plasma were related to the increased risk of BPD [5]. Studies dealing with the bronchoalveolar lavage fluid of children with BPD have also revealed some proteins that are potentially involved in the pathomechanism of BPD, like matrix metalloproteinase-3 [6]. However, blood samples remain the preferable, and best available, material for screening for markers and obtaining additional information regarding the course of the disease.

In our previous publications, we described the comparison between abundances of all plasma proteins from prematurely born children with different gestational ages, both from cord blood as well as at the 36th postmenstrual week (36 PMA) [7–9]. The articles presented that proteome differences are highly gestational age-dependent. Recently, we also published data describing the differences in plasma protein abundances in prematurely born infants with and without retinopathy of prematurity (ROP) [10].

In this study, we aimed to identify potential BPD plasma biomarkers and to provide a more molecular-based understanding of BPD, by comparing a proteome profile at two time points (at birth and at the 36 PMA) in groups of infants with and without BPD. However, according to our previous observations, the level of prematurity has a fundamental influence on the plasma protein quantitative changes [7–9]. Therefore, we performed a standardization of the obtained results for the gestational age.

## Methods

In this paper, we analyzed data obtained from a multicenter study to explore proteome in preterm infants. The study was approved by the Jagiellonian University Bioethical Committee and adheres to the tenets of the Declaration of Helsinki.

### Enrolled patients

We investigated all newborns with a gestational age of ≤30 weeks, consecutively enrolled between September 1st 2013 and November 30th 2015 at the Warsaw Medical University Neonatal Intensive Care Unit (NICU). Parents signed the informed consent antenatally.

### Blood sampling

After birth we collected cord blood samples from all study participants. A second blood sample (peripheral venous blood) was taken at 36 PMA. The plasma samples were further used for Combinatorial Peptide Ligand Libraries- isobaric Tag for Relative and Absolute Quantitation (CPLL-iTRAQ) quantitative analysis as previously described [8, 10].

### Proteome analysis

ProteoMiner beads (CPLL beads, Bio-Rad, Hercules, CA) were used for the enrichment procedure, optimized with reference to previously published protocols [11, 12]. The quantitative analysis was performed by iTRAQ method (Sciex, Framingham, MA). Samples were enriched, trypsin-digested, randomly assigned to iTRAQ reagents, labeled according to the manufacturer instructions and, finally, combined to the corresponding 8plex assemblies. For data normalization, each 8plex assembly contained an internal common reference generated by combining equal amounts of protein from all the samples included in the measurements. Next, labeled peptides were fractionated off-line by Strong Cation Exchange (SCX) chromatography on SCX Macrospin columns (Harvard Apparatus), collecting by centrifugation (2000×g, 1 min) the flow-through fraction and 11 consecutive injections of the eluent buffer, comprising 5, 10, 20, 40, 60, 80, 100, 150, 200, 300, and 500 mM ammonium acetate in 5% ACN and 0.1% FA. Thus, the labeled peptides from each 8plex assembly were distributed across 12 SCX fractions. Each fraction was then separated by reversed-phase liquid chromatography and applied on-line to a Velos Pro (Thermo Scientific, Waltham, MA) mass spectrometer through a nano-electrospray ion source. Labeled peptides were injected onto a PepMap100 RP C18 75 μm i.d. × 15 cm column (Thermo Scientific, Waltham, MA) via a trap column PepMap100 RP C18 75 μm i.d. × 2 cm column (Thermo Scientific, Waltham, MA). Each peptide fraction was separated using a 65 min 7 to 45% B phase linear gradient (A phase - 2% ACN and 0.1% FA; B phase - 80% ACN and 0.1% FA) operating at a flow rate of 300 nL/min on an UltiMate 3000 HPLC system (Thermo Scientific, Waltham, MA). Spectra were collected in full scan mode (400–1500 Da), followed by five pairs of Collisional-Induced Dissociation (CID) and Higher Energy Collisional Dissociation (HCD) tandem mass spectrometry (MS/MS) scans of the five most intense precursor ions from the survey full scan and, subsequently, merged to hybrid HCD-CID spectra by EasierMGF software [13]. These were analyzed by the X!Tandem (The GPM Organization) [14] and Comet [15] search engines, statistically validated with PeptideProphet and integrated with iProphet [16] under the Trans-Proteomic Pipeline (TPP) suite of software (Institute for Systems Biology, Seattle, WA, USA) [17]. The Peptide False Discovery Rate (FDR) was estimated by Mayu [18] (TPP) and peptide identifications with an FDR below 1% were considered to be correct matches. Imputation of the missing values in peptide abundances was performed in a MaxQuant environment [19] on the log_2_-transformed normalized iTRAQ, which reports intensities with a criterion of at least 75% of the values present for a peptide in the dataset by drawing the values from the normal distribution, with parameters optimized to mimic a typical low abundance measurement. DanteR software [20] was used for protein quantitation and the statistical analysis of iTRAQ-labeled peptides. ANOVA was performed at the peptide level using a linear model with the Benjamini and Hochberg False Discovery Rate (FDR) correction used to adjust *p*-values. Protein fold change was reported as a median value of corresponding unique peptides.

### Monitoring during hospitalization

All the subjects enrolled in the study underwent careful clinical monitoring for symptoms of BPD, as the standard of care. The presence and severity of BPD were assessed according to the NICHD diagnostic criteria at 36 week postmenstrual age or discharge to home, whichever came first [21]. BPD was recognized in a child treated with oxygen > 21% for at least 28 days plus: for mild BPD – breathing room air at 36 week postmenstrual age or discharge to home, whichever came first; for moderate BPD – requiring < 30% oxygen at 36 week postmenstrual age or discharge to home, whichever came first; for severe BPD – requiring ≥30% oxygen and/or positive pressure (positive pressure ventilation or nasal continuous positive air pressure) at 36 week postmenstrual age or discharge to home, whichever came first.

### Division into groups

Patients who developed BPD (any level of severity) were included into the BPD(+) group, whereas patients without diagnosed BPD formed the BPD(−) group.

### Data collection

The patient’s data, involving perinatal history, hospitalization course and the incidence of prematurity complications with special regard to the occurrence of BPD, were simultaneously collected.

### Justification of sample size

A formal sample size calculation was not performed so as to allow the realization of a hypothesis generating study. The approximate sample size was based on the calculations for the main study [7]. Briefly, the basic goal of the main project was to compare protein abundance levels between groups with a different degree of maturity. The power analysis (https://www.dssresearch.com/resources/calculators/) indicated that with *n* = 19 patients in each preterm group, the estimated power of the study to validate the measured fold-change at the level of 1.15 was 0.98 (*p* = 0.05). Therefore, *n* = 19 patients were enrolled to each preterm subgroup. Using the calculation mentioned above we can state that the power of the present study is at least as in the publication mentioned above - we can detect at least a 1.15 fold change protein abundance between BPD(−) and BPD(+) patients.

### Statistical analysis

Differences between the groups were compared using a Wilcoxon-test (one-way, Chi^2^ approximation) or Pearson-Chi^2^-test, as appropriate. Studied groups were compared directly (crude data) and after standardization for gestational age (adjusted data). A linear model was fitted to the protein abundances for each protein, and t-tests and F-statistics were computed for each contrast - group indicator and gestational age. For each protein that was found to have a different concentration between the groups, i.e., that had a false discovery rate–adjusted *p*-value < 0.05 in the first part of the analysis, it was tested whether this presence was explained by the group indicator and/or by gestational age, using logistic-regression analysis. For statistical analysis, an SPSS software package (IBM SPSS Statistics for iOS, Version 24.0. Armonk, NY: IBM Corp.) was applied.

## Results

**Fig. 1:**
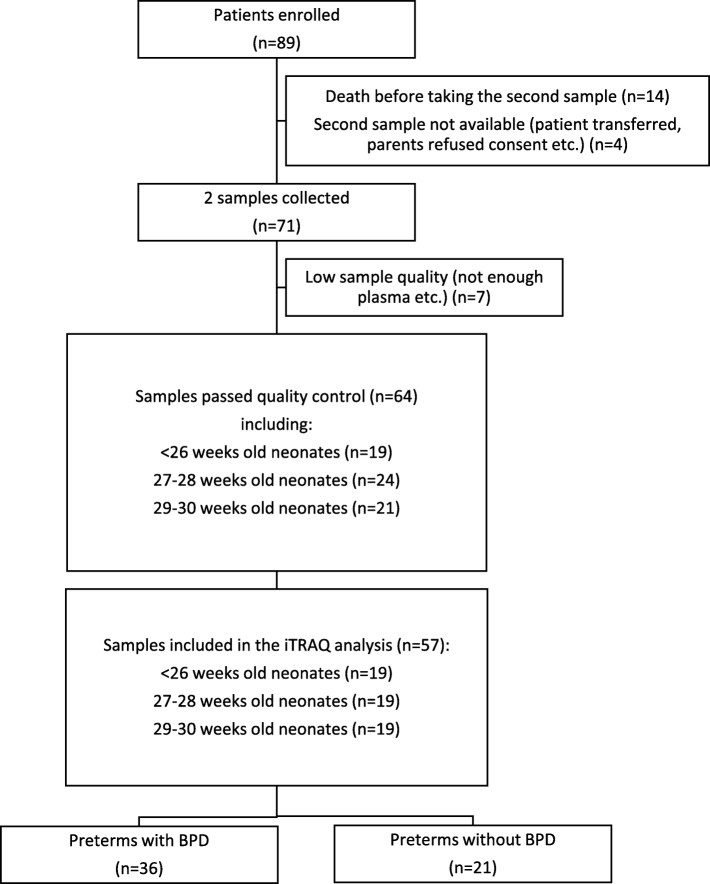
Flowchart showing samples included in the iTRAQ analysis

Fifty-seven preterm newborns were included in the study. Two time points of blood drawing resulted in a total of 114 plasma samples for proteomic analysis. During their hospitalization in the NICU, 36 infants developing BPD formed the BPD(+) group and 21 infants not meeting the diagnostic criteria for BPD formed the BPD(−) group (Fig. 1).

The overall characteristics of the cohort and the differences in selected variables across the analyzed groups are shown in Table 1.

**Table 1:** Comparison of selected demographic variables and hospitalization data of the patients in the studied groups

	BPD(−) group *n* = 21	BPD(+) group *n* = 36	*p*
Gestational age [weeks], median [Q25; Q75]	29 [28; 29]	26 [24.25; 28]	**< 0.0001** ^**a**^
Birthweight [g], median [Q25; Q75]	1145 [990; 1415]	870 [732.5; 1000]	**0.0002** ^**a**^
Female gender; *n* (%)	13 (62%)	25 (69%)	0.5602 ^b^
Antenatal steroids; *n* (%)	15 (71%)	29 (81%)	0.4283 ^b^
Sepsis; *n* (%)	1 (5%)	2 (6%)	0.8970 ^b^
Intraventricular hemorrhage grade 3/4; *n* (%)	0 (0%)	2 (6%)	0.2715 ^b^
Patent ductus arteriosus; *n* (%)	3 (14%)	23 (64%)	**0.0003** ^**b**^
Pneumonia; *n* (%)	0 (0%)	11 (31%)	**0.0042** ^**b**^
Necrotizing enterocolitis; *n* (%)	0 (0%)	5 (14%)	0.0695 ^b^
Bronchopulmonary dysplasia; *n* (%)	0 (0%)	36 (100%)	**< 0.0001** ^**b**^
- mild		29 (81%)	
- moderate		7 (19%)	
- severe		0 (0%)	
Retinopathy of prematurity; *n* (%)	4 (19%)	24 (67%)	**0.0005** ^**b**^
- requiring laser coagulation	0 (0%)	5 (14%)	0.0738 ^b^

^a^ Wilcoxon-test (one-way, Chi^2^ approximation); ^b^ Pearson-Chi^2^-test

Children, developing BPD during hospitalization, were characterized by a lower gestational age and birthweight, and that they also developed pneumonia, patent ductus arteriosus and ROP more often. Among the children with BPD, in 29 the mild form of BPD was recognized, whereas seven of them presented moderate BPD and none severe BPD. This sub-group with moderate BPD generally consisted of the most immature infants with a median gestational age of 24 weeks [24; 26], birthweight – 700 g [670; 730], female gender – 7 (100%), prenatal steroids – 5 (71%), sepsis – 1 (14%), intraventricular hemorrhage grade 3/4–1 (14%), patent ductus arteriosus - 7 (100%), pneumonia - 3 (43%), necrotizing enterocolitis - 1 (14%), ROP - 6 (86%), ROP requiring laser photocoagulation - 2 (29%).

In the first stage, we analyzed the concentrations of proteins in CPLL-enriched plasma obtained from the cord blood. The abundance level of 33 of the proteins for the group who, during hospitalization, developed BPD were significantly different when compared to preterm infants without this complication (Table 2).

**Table 2:** The baseline differences in cord blood plasma proteome among infants who, subsequently during hospitalization at NICU, developed BPD vs. those who did not develop this condition, before and after adjustment for gestational age

UniProt Protein Name	UniProt accession	Ratio	*p*-value for multiple comparisons	Ratio adjusted for GA	p adjusted for GA and multiple comparisons
Afamin	P43652	0.924591928	0.034838518	0.815	**0.0105**
Alpha-1-acid glycoprotein 2	P19652	1.191226628	0.041937129	0.941	0.521
Alpha-1-antichymotrypsin	P01011	1.283782192	2.97E-07	0.942	0.851
Alpha-1-antitrypsin	P01009	1.116832587	3.96E-05	0.948	0.781
Alpha-1B-glycoprotein	P04217	1.098381007	0.037425994	0.906	0.562
Alpha-2-HS-glycoprotein	P02765	1.122196808	0.002698155	0.941	0.527
Alpha-2-macroglobulin	P01023	0.870825049	3.32E-10	0.814	0.346
Alpha-fetoprotein	P02771	1.116445176	0.000580535	0.565	0.151
Angiotensinogen	P01019	1.1105723	0.009877406	0.877	0.561
Apolipoprotein A-I	P02647	0.898460938	1.42E-05	0.755	0.055
Apolipoprotein A-IV	P06727	0.916911033	0.001791272	0.911	0.551
Apolipoprotein C-II	P02655	1.212022332	9.25E-07	1.12	0.689
Carboxypeptidase N subunit 2	P22792	0.869027963	0.000698996	0.694	**0.048**
Corticosteroid-binding globulin	P08185	1.110918618	0.022913597	0.992	0.981
Galectin-3-binding protein	Q08380	0.873539268	0.000474608	0.804	0.236
Gelsolin	P06396	0.926610554	0.010030638	0.935	**0.044**
Haptoglobin	P00738	1.716109241	1.31E-09	1.14	0.804
Hemoglobin subunit alpha	P69905	0.819939091	0.007337795	0.631	0.425
Hemoglobin subunit beta	P68871	0.758775176	6.56E-07	0.627	0.272
Hemoglobin subunit gamma-1	P69891	0.734345636	2.21E-06	0.613	0.342
Hemoglobin subunit gamma-2	P69892	0.818679705	0.003576161	0.581	0.423
Hemopexin	P02790	1.355543556	3.96E-11	0.96	0.816
Immunoglobulin heavy constant gamma 1	P01857	0.845180753	0.004295591	0.889	0.668
Immunoglobulin heavy constant gamma 3	P01860	0.894240288	0.019094856	0.817	0.381
Immunoglobulin kappa constant	P01834	0.884133158	0.02568417	0.914	0.65
Inter-alpha-trypsin inhibitor heavy chain H2	P19823	0.910909468	7.64E-05	0.886	0.451
Inter-alpha-trypsin inhibitor heavy chain H3	Q06033	1.10230461	0.005007488	0.969	0.871
Inter-alpha-trypsin inhibitor heavy chain H4	Q14624	1.130490456	0.008325306	0.952	0.792
Leucine-rich alpha-2-glycoprotein	P02750	1.156790051	0.007822803	1.036	0.687
Lumican	P51884	0.90816737	0.001983702	0.946	0.687
Lymphatic vessel endothelial hyaluronic acid receptor 1	Q9Y5Y7	1.149880659	0.018921004	1.134	0.381
Plasma protease C1 inhibitor	P05155	1.153150546	1.27E-06	1.012	0.939
Vitronectin	P04004	1.152815567	9.09E-05	0.824	0.44

The quantitative comparisons between samples collected at 36 PMA from BPD(+) and BPD(−) groups revealed a significantly different abundance level of 27 proteins (Table 3).

**Table 3:** Differences in plasma proteome among infants who developed BPD vs. those who did not develop this disease, before and after adjustment for gestational age, assessed at 36 PMA

UniProt Protein Name	UniProt accession	Ratio	*p* value for multiple comparisons	Ratio adjusted for GA	p adjusted for GA and multiple comparisons
Alpha-2-antiplasmin	P08697	0.902954091	0.009571493	1.07	0.652
Alpha-fetoprotein	P02771	1.205017172	1.48E-06	1.073	0.651
Apolipoprotein A-I	P02647	0.921011303	0.000666248	0.97	0.851
Apolipoprotein A-IV	P06727	0.929138146	0.00183834	0.91	0.771
Carboxypeptidase N subunit 2	P22792	0.913686152	0.034019428	0.859	0.343
CD44 antigen	P16070	0.857799986	0.001787237	1.111	0.514
Clusterin	P10909	1.134578843	0.000701756	1.043	0.733
Complement C1r subcomponent	P00736	0.900163532	0.040257434	0.976	0.884
Complement C1s subcomponent	P09871	0.858210146	2.42E-06	1.018	0.911
Corticosteroid-binding globulin	P08185	1.097266083	0.048487179	1.037	0.707
Fibrinogen gamma chain	P02679	1.073909534	0.049721103	1.154	0.309
Galectin-3-binding protein	Q08380	0.859983677	9.90E-05	0.786	**0.025**
Gelsolin	P06396	0.886021433	8.90E-05	0.953	0.708
Haptoglobin	P00738	1.484385427	5.56E-05	1.286	0.478
Hemoglobin subunit beta	P68871	1.250987956	0.000612418	1.114	0.329
Hemoglobin subunit gamma-1	P69891	0.804233584	0.007778202	0.776	**0.038**
Immunoglobulin heavy constant gamma 1	P01857	0.854508568	0.017674648	1.22	0.369
Immunoglobulin heavy constant gamma 3	P01860	0.889487254	0.027804083	0.982	0.9583
Immunoglobulin lambda constant 2; Immunoglobulin lambda constant 3	P0CG06	0.762467463	0.040257434	0.919	0.543
Immunoglobulin lambda-like polypeptide 5	B9A064	0.881285593	0.029037441	1.024	0.811
Inter-alpha-trypsin inhibitor heavy chain H2	P19823	0.924508903	0.001365124	0.988	0.924
Kininogen-1	P01042	1.058078701	0.03628071	1.134	0.319
Leucine-rich alpha-2-glycoprotein	P02750	1.166917588	0.002032426	1.224	0.278
Osteopontin	P10451	0.872885059	0.027176592	0.982	0.92
Serotransferrin	P02787	1.123191657	4.19E-06	1.284	**0.023**
Transthyretin	P02766	1.183233993	0.017525513	1.122	0.432
Vitronectin	P04004	1.084990139	0.035870579	0.951	0.717

After standardization for gestational age, children with BPD were characterized by a decreased abundance of afamin, gelsolin and carboxypeptidase N subunit 2, together with a borderline, decreased apolipoprotein A-I level in cord blood. They also had a decreased galectin-3 binding protein and hemoglobin subunit gamma-1 level, as well as an increased serotransferrin level in plasma at the 36 PMA (Tables 2 and 3).

## Discussion

Our study presents the results of plasma proteome analysis of infants ≤30 gestational weeks who developed or omitted BPD. Our findings support previous literature by showing that a lower gestational age and birth weight correlate with a higher risk of developing BPD [2]. It is noteworthy that the BPD(+) group developed ROP more often, which may be explained by the fact that these prematurity complications share common etiologic factors [22]. Moreover, we identified three proteins, whose decreased abundance in cord blood plasma separates children with and without the risk of subsequently developing BPD. Furthermore, we also found that at the 36 PMA, children with diagnosed BPD had a different plasma level of the other three proteins, pointing to additional complications that children with BPD are exposed to.

Afamin is a vitamin E-binding serum glycoprotein, with the highest affinity to α- and γ-tocopherol isoforms [23, 24]. Moreover, it is involved in anti-apoptotic cellular processes related to oxidative stress [25]. Afamin in the bloodstream is partially associated with apolipoprotein A-I (ApoA1)-containing high density lipoprotein subfractions [24]. As evidenced by the iTRAQ method, both proteins share the same concentration shift in cord blood samples. Of note, lipoproteins are considered main blood carrier vehicles for tocopherols [26]. The role of oxidative stress is considered in the complex pathogenesis of BPD, and current studies report a protective role of antioxidant melatonin against hyperoxic lung injury both in the rodents [27] and preterm neonates [28, 29]. Tocopherols are one of the most potent antioxidants, and vitamin E deficiency has been associated with an increased risk of BPD [30, 31]. We are tempted to speculate that a decreased abundance of afamin and ApoA1 in cord blood may indicate disturbances in tocopherol availability and, resulting from this, a lower potential to neutralize oxidative damage present at the beginning of life of premature infants. Therefore, there is an increased risk of them developing chronic lung injury. Several studies have shown a decreased level of vitamin E in prematurely born neonates shortly after birth [32]. Unfortunately, until now, clinical studies aimed at reducing the frequency of BPD by the supplementation of vitamin E have not brought the expected favorable results [33]. Our finding may support the idea of re-reviewing the hitherto knowledge about the connection between vitamin E and BPD, and the possible methods of preventing or alleviating this severe complication of prematurity, by obtaining the adequate level of appropriate isoform of this antioxidant in the maternal/infant organism [34]. We also believe that it is worth investigating whether the lower level of vitamin E carrier proteins might decrease the bioavailability of vitamin E, despite supplementation.

Carboxypeptidase N (CPN) consists of two small (CPN1) and two large subunits (CPN2). It can indirectly modulate immune response by cleaving amino acids (lysine and arginine) from the carboxy-terminus of selected proteins [35]. CPN reduces the activity of kallidin, involved in acute and chronic phase of inflammatory response [36], and inactivates anaphylatoxins [37]. Additionally, it supports the production of nitric oxide [35]. We postulate, that the lower abundance of CPN observed in the cord blood of neonates that develop BPD, might be related to the impaired mechanisms discussed above.

Gelsolin is involved in the regulation of cytoskeletal formation [38]. Its deficiency has been linked with blunted responses to stress conditions of blood platelets, neutrophils and fibroblasts, resulting in impaired hemostasis, inflammation and wound healing [39]. Of note, an insufficiency of gelsolin in rodents has also been shown to cause increased permeability of lung vessels, suggesting that gelsolin is important for the local response to lung injury [38].

Galectin-3-binding protein (Gal-3BP) is a significant component of innate immunity [40], that modulates the secretion of several cytokines [41] and increases the amount of surface antigens important for immune responses [42–44]. Additionally, it possesses antiviral properties [45], and in case of bacterial infection it suppresses the lipopolysaccharide (LPS)-induced secretion of cytokines [46], as well as production of reactive oxygen species [47]. We postulate, that the lower abundance of this protein at the 36 PMA in infants with BPD, might be related to the observed decreased resistance to infections [48–50], moreover, it may exacerbate infections affecting children with BPD-damaged lungs. However, whether a lower amount of this protein is also present beyond an early infancy period requires further research.

Fetal hemoglobin (HbF), consisting of two alpha and two gamma chains, is gradually replaced by adult variants (two alpha and two beta chains) late in infancy. HbF is characterized by a greater affinity for oxygen and the ability to saturate with oxygen at a lower partial pressure. Presumably a lower abundance of Hb subunit gamma-1 in children with BPD may relate to the more frequent former transfusions of adult packed red blood cells in this group of patients. However, our results are contrary to the study of Bard and Prosmanne, who observed an increased production of HbF in children with BPD during the first year of life, probably as a result of an erythropoietic response to hypoxemia [51].

Serotransferrin transports iron from sites of storage to regions of iron metabolism [52]. Its level increases in case of iron deficiency. As ferropenia and anemia are frequent in children with chronic respiratory diseases [53], we suggest that our finding of an increased abundance of serotransferrin in children with BPD may be connected with this observation. Moreover, serotransferrin is also an acute phase protein that may indicate a low-grade inflammation present at 36 PMA in children from the BPD group.

Limitations: The specific character of the studied population might have influenced the results that were obtained; namely that they were biased towards a statistically significant difference. The patient population may not be applicable to other NICUs (for example: the BPD rate), so our results may not be easily generalizable. The BPD group consisted mainly of children with a mild form of BPD, which may raise doubts about whether it is a chronic lung disease or only a more severe respiratory distress syndrome – a lack of children with severe BPD is one of the limitations of this research. Due to the small size of the subgroup with moderate BPD, we did not carry out a separate analysis of this infants. Moreover, due to the nature of our study, there may be a potential collider bias in controlling for gestational age when low gestational age is highly associated with preterm birth, which is linked with BPD and potentially pathophysiologic factors. Also, we cannot exclude a potential influence of some factors such as patent ductus arteriosus or pneumonia on the observed differences in protein abundances between the BPD and non-BPD groups. Additionally, the validation of proteomic results by another method would strengthen the iTRAQ quantitation results. It must be emphasized, that the challenge in our study was the in-depth screening insight into changes in plasma proteins, which inevitably imposes the requirements of a large amount of the sample and specific preparation protocols to be used with the multidimensional chromatography - mass spectrometry analysis. However, this unique methodology alone offers the possibility to overcome the issue of the specific dynamic range of protein concentrations in the plasma for the unbiased, untargeted proteome-wide quantitative measurements. Thus, we present here a blueprint of plasma proteome changes in preterm neonates for further, targeted studies, designed to unravel the influence of the individual proteins on BPD development and progression, as well as for their biomarker utility.

## Conclusions

Our study reveals that BPD development is associated with the plasma proteome changes in preterm infants, adding additional evidence for their possible involvement in disturbances of vitamin E availability and impaired immunological processes in the progression of neonate pulmonary complications. Moreover, it also points to the differences in proteins related to infection resistance and maintaining adequate hematocrit in children diagnosed with BPD.

## Data Availability

The datasets used and/or analyzed during the current study are available from the corresponding author on reasonable request.

## Abbreviations

ApoA1: Apolipoprotein A-I
BPD: Bronchopulmonary dysplasia
CID: Collisional-Induced Dissociation
CPLL: Combinatorial peptide ligand libraries
CPN: Carboxypeptidase N
FDR: False Discovery Rate
Gal-3BP: Galectin-3-binding protein
HbF: Fetal hemoglobin
HCD: Higher Energy Collisional Dissociation
iTRAQ: Isobaric tag for relative and absolute quantitation
LPS: Lipopolysaccharide
MS/MS: Tandem mass spectrometry
NICU: Neonatal intensive care unit
PMA: Postmenstrual week
ROP: Retinopathy of prematurity
SCX: Strong Cation Exchange
TPP: Trans-Proteomic Pipeline

## References

[1] Bhandari A, Panitch H (2018). An update on the post-NICU discharge management of bronchopulmonary dysplasia. Semin Perinatol.

[2] Jobe AH (2016). Mechanisms of lung injury and bronchopulmonary dysplasia. Am J Perinatol.

[3] Savani RC (2018). Modulators of inflammation in bronchopulmonary dysplasia. Semin Perinatol.

[4] Stoll BJ, Hansen NI, Bell EF, Walsh MC, Carlo WA, Shankaran S (2015). Trends in care practices, morbidity, and mortality of extremely preterm neonates, 1993-2012. JAMA.

[5] Förster K, Sass S, Ehrhardt H, Mous DS, Rottier RJ, Oak P (2018). Early identification of bronchopulmonary dysplasia using novel biomarkers by proteomic screening. Am J Respir Crit Care Med.

[6] Vento G, Tirone C, Lulli P, Capoluongo E, Ameglio F, Lozzi S (2009). Bronchoalveolar lavage fluid peptidomics suggests a possible matrix metalloproteinase-3 role in bronchopulmonary dysplasia. Intensive Care Med.

[7] Suski M, Bokiniec R, Szwarc-Duma M, Madej J, Bujak-Giżycka B, Borszewska-Kornacka MK (2018). Plasma proteome changes in cord blood samples from preterm infants. J Perinatol.

[8] Suski M, Bokiniec R, Szwarc-Duma M, Madej J, Bujak-Giżycka B, Kwinta P (2018). Prospective plasma proteome changes in preterm infants with different gestational ages. Pediatr Res.

[9] Kwinta P, Bokiniec R, Bik-Multanowski M, Gunther CC, Grabowska A, Książek T (2017). Comparison of whole genome expression profile between preterm and full-term newborns. Ginekol Pol.

[10] Zasada M, Suski M, Bokiniec R, Szwarc-Duma M, Borszewska-Kornacka MK, Madej J (2018). An iTRAQ-based quantitative proteomic analysis of plasma proteins in preterm newborns with retinopathy of prematurity. Invest Ophthalmol Vis Sci.

[11] Candiano G, Dimuccio V, Bruschi M, Santucci L, Gusmano R, Boschetti E (2009). Combinatorial peptide ligand libraries for urine proteome analysis: investigation of different elution systems. Electrophoresis.

[12] Fasoli E, Farinazzo A, Sun CJ, Kravchuk AV, Guerrier L, Fortis F (2010). Interaction among proteins and peptide libraries in proteome analysis: pH involvement for a larger capture of species. J Proteome.

[13] Gallardo Ó, Ovelleiro D, Gay M, Carrascal M, Abian J (2014). A collection of open source applications for mass spectrometry data mining. Proteomics.

[14] Craig R, Beavis RC (2004). TANDEM: matching proteins with tandem mass spectra. Bioinformatics.

[15] Eng JK, Jahan TA, Hoopmann MR (2013). Comet: an open-source MS/MS sequence database search tool. Proteomics.

[16] Shteynberg D, Deutsch EW, Lam H, Eng JK, Sun Z, Tasman N (2011). iProphet: multi-level integrative analysis of shotgun proteomic data improves peptide and protein identification rates and error estimates. Mol Cell Proteomics.

[17] Deutsch EW, Mendoza L, Shteynberg D, Farrah T, Lam H, Tasman N (2010). A guided tour of the trans-proteomic pipeline. Proteomics.

[18] Reiter L, Claassen M, Schrimpf SP, Jovanovic M, Schmidt A, Buhmann JM (2009). Protein identification false discovery rates for very large proteomics data sets generated by tandem mass spectrometry. Mol Cell Proteomics.

[19] Cox J, Mann M (2008). MaxQuant enables high peptide identification rates, individualized p.p.b.-range mass accuracies and proteome-wide protein quantification. Nat Biotechnol.

[20] Taverner T, Karpievitch YV, Polpitiya AD, Brown JN, Dabney AR, Anderson GA (2012). DanteR: an extensible R-based tool for quantitative analysis of -omics data. Bioinformatics..

[21] Jobe AH, Bancalari E (2001). Bronchopulmonary dysplasia. Am J Respir Crit Care Med.

[22] Saugstad OD (1988). Hypoxanthine as an indicator of hypoxia: its role in health and disease through free radical production. Pediatr Res.

[23] Voegele AF, Jerković L, Wellenzohn B, Eller P, Kronenberg F, Liedl KR (2002). Characterization of the vitamin E-binding properties of human plasma afamin. Biochemistry.

[24] Jerkovic L, Voegele AF, Chwatal S, Kronenberg F, Radcliffe CM, Wormald MR (2005). Afamin is a novel human vitamin E-binding glycoprotein characterization and in vitro expression. J Proteome Res.

[25] Heiser M, et al. Vitamin E binding protein A famin Protects Neuronal Cells in vitro. In: Jellinger KA, Schmidt R, Windisch M. (eds) Ageing and Dementia Current and Future Concepts. Springer, Vienna. Journal of Neural Transmission. 2002;62:337-45. 10.1007/978-3-7091-6139-5_32 12456077

[26] Herrera E, Barbas C (2001). Vitamin E: action, metabolism and perspectives. J Physiol Biochem.

[27] Suleymanoglu S, Cekmez F, Cetinkaya M, Tayman C, Canpolat FE, Kafa IM (2014). Protective effects of melatonin therapy in model for neonatal hyperoxic lung injury. Altern Ther Health Med.

[28] Gitto E, Reiter RJ, Cordaro SP, La Rosa M, Chiurazzi P, Trimarchi G (2004). Oxidative and inflammatory parameters in respiratory distress syndrome of preterm newborns: beneficial effects of melatonin. Am J Perinatol.

[29] Gitto E, Reiter RJ, Amodio A, Romeo C, Cuzzocrea E, Sabatino G (2004). Early indicators of chronic lung disease in preterm infants with respiratory distress syndrome and their inhibition by melatonin. J Pineal Res.

[30] Falciglia HS, Ginn-Pease ME, Falciglia GA, Lubin AH, Frank DJ, Chang W (1988). Vitamin E and selenium levels of premature infants with severe respiratory distress syndrome and bronchopulmonary dysplasia. J Pediatr Perinat Nutr.

[31] Falciglia HS, Johnson JR, Sullivan J, Hall CF, Miller JD, Riechmann GC (2003). Role of antioxidant nutrients and lipid peroxidation in premature infants with respiratory distress syndrome and bronchopulmonary dysplasia. Am J Perinatol.

[32] Wu SC, Chou YH (2001). Measurement of serum vitamin E isomers in fullterm and preterm infants. Chang Gung Med J.

[33] Brion LP, Bell EF, Raghuveer TS. Vitamin E supplementation for prevention of morbidity and mortality in preterm infants. Cochrane Database of Systematic Reviews. 2003;(4). Art. No.: CD003665. 10.1002/14651858.CD003665. 10.1002/14651858.CD003665 PMC8725195 12917978

[34] Stone CA, McEvoy CT, Aschner JL, Kirk A, Rosas-Salazar C, Cook-Mills JM (2018). Update on vitamin E and its potential role in preventing or treating bronchopulmonary dysplasia. Neonatology.

[35] Matthews KW, Mueller-Ortiz SL, Wetsel RA (2004). Carboxypeptidase N: a pleiotropic regulator of inflammation. Mol Immunol.

[36] Couture R, Harrisson M, Vianna RM, Cloutier F (2001). Kinin receptors in pain and inflammation. Eur J Pharmacol.

[37] Bokisch VA, Müller-Eberhard HJ (1970). Anaphylatoxin inactivator of human plasma: its isolation and characterization as a carboxypeptidase. J Clin Invest.

[38] Becker PM, Kazi AA, Wadgaonkar R, Pearse DB, Kwiatkowski D, Garcia JG (2003). Pulmonary vascular permeability and ischemic injury in gelsolin-deficient mice. Am J Respir Cell Mol Biol.

[39] Witke W, Sharpe AH, Hartwig JH, Azuma T, Stossel TP, Kwiatkowski DJ (1995). Hemostatic, inflammatory, and fibroblast responses are blunted in mice lacking gelsolin. Cell.

[40] Loimaranta V, Hepojoki J, Laaksoaho O, Pulliainen AT (2018). Galectin-3-binding protein: a multitask glycoprotein with innate immunity functions in viral and bacterial infections. J Leukoc Biol.

[41] Kalayci O, Birben E, Tinari N, Oguma T, Iacobelli S, Lilly CM (2004). Role of 90K protein in asthma and TH2-type cytokine expression. Ann Allergy Asthma Immunol.

[42] Powell TJ, Schreck R, McCall M, Hui T, Rice A, App H (1995). A tumor-derived protein which provides T-cell costimulation through accessory cell activation. J Immunother Emphasis Tumor Immunol.

[43] Grassadonia A, Tinari N, Fiorentino B, Suzuki K, Nakazato M, De Tursi M (2004). The 90K protein increases major histocompatibility complex class I expression and is regulated by hormones, gamma-interferon, and double-strand polynucleotides. Endocrinology.

[44] Natoli C, Iacobelli S, Kohn L (1996). The immune stimulatory protein 90K increases major histocompatibility complex class I expression in a human breast cancer cell line. Biochem Biophys Res Commun.

[45] Denard J, Beley C, Kotin R, Lai-Kuen R, Blot S, Leh H (2012). Human galectin 3 binding protein interacts with recombinant adeno-associated virus type 6. J Virol.

[46] Trahey M, Weissman IL (1999). Cyclophilin C-associated protein: a normal secreted glycoprotein that down-modulates endotoxin and proinflammatory responses in vivo. Proc Natl Acad Sci U S A.

[47] Läubli H, Alisson-Silva F, Stanczak MA, Siddiqui SS, Deng L, Verhagen A (2014). Lectin galactoside-binding soluble 3 binding protein (LGALS3BP) is a tumor-associated immunomodulatory ligand for CD33-related Siglecs. J Biol Chem.

[48] Landry JS, Chan T, Lands L, Menzies D (2011). Long-term impact of bronchopulmonary dysplasia on pulmonary function. Can Respir J.

[49] Carpenter TC, Stenmark KR (2004). Predisposition of infants with chronic lung disease to respiratory syncytial virus-induced respiratory failure: a vascular hypothesis. Pediatr Infect Dis J.

[50] Caskey S, Gough A, Rowan S, Gillespie S, Clarke J, Riley M (2016). Structural and functional lung impairment in adult survivors of bronchopulmonary dysplasia. Ann Am Thorac Soc.

[51] Bard H, Prosmanne J (1990). Elevated levels of fetal hemoglobin synthesis in infants with bronchopulmonary dysplasia. Pediatrics.

[52] MacGillivray RT, Moore SA, Chen J, Anderson BF, Baker H, Luo Y (1998). Two high-resolution crystal structures of the recombinant N-lobe of human transferrin reveal a structural change implicated in iron release. Biochemistry.

[53] Barja S, Capo E, Briceño L, Jakubson L, Méndez M, Becker A (2013). Anemia and iron deficiency in children with chronic respiratory diseases. Nutr Hosp.

